# Sirtuin modulators control reactive gliosis in an *in vitro* model of Alzheimer’s disease

**DOI:** 10.3389/fphar.2014.00089

**Published:** 2014-05-13

**Authors:** Caterina Scuderi, Claudia Stecca, Maria R. Bronzuoli, Dante Rotili, Sergio Valente, Antonello Mai, Luca Steardo

**Affiliations:** ^1^Vittorio Erspamer School of Physiology and Pharmacology, SAPIENZA University of RomeRome, Italy; ^2^Department of Drug Chemistry and Technologies, SAPIENZA University of RomeRome, Italy; ^3^Institute Pasteur – Cenci Bolognetti Foundation, SAPIENZA University of RomeRome, Italy

**Keywords:** resveratrol, AGK-2, sirtuins, beta-amyloid, astrocyte, reactive gliosis, Alzheimer’s disease

## Abstract

Among neurodegenerative disorders, Alzheimer’s disease (AD) represents the most common cause of dementia in the elderly. Several genetic and environmental factors have been identified; however, aging represents the most important risk factor in the development of AD. To date, no effective treatments to prevent or slow this dementia are available. Sirtuins (SIRTs) are a family of NAD^+^-dependent enzymes, implicated in the control of a variety of biological processes that have the potential to modulate neurodegeneration. Here we tested the hypothesis that activation of SIRT1 or inhibition of SIRT2 would prevent reactive gliosis which is considered one of the most important hallmark of AD. Primary rat astrocytes were activated with beta amyloid 1-42 (Aβ 1-42) and treated with resveratrol (RSV) or AGK-2, a SIRT1 activator and a SIRT2-selective inhibitor, respectively. Results showed that both RSV and AGK-2 were able to reduce astrocyte activation as well as the production of pro-inflammatory mediators. These data disclose novel findings about the therapeutic potential of SIRT modulators, and suggest novel strategies for AD treatment.

## INTRODUCTION

Alzheimer’s disease (AD) represents one of the major health concern and it is a research priority since there is a pressing need to develop new agents to prevent or treat it. A part of the progressive deposition of beta amyloid peptide (Aβ) and accumulation of phosphorylated tau, several other alterations occur in AD brain, all concurring to neuronal loss. Among these, growing interest has been attracted by the role of inflammation in the onset and progression of this disorder. In fact, senile plaques and neurofibrillary tangles (which are considered the more characteristic hallmarks of AD) co-localize with activated astrocytes, suggesting for these cells a key role in the pathogenesis of AD (Meda et al., 2001; Craft et al., 2006). Along this line, in several experimental models it has been demonstrated that Aβ peptide fragments markedly alter astrocytes functions. This process is accompanied with a noticeable neuroinflammatory response, accounting for the synthesis of different cytokines and pro-inflammatory mediators which amplify neuropathological damage (Mrak and Griffin, 2001; Caricasole et al., 2003; Tuppo and Arias, 2005; Griffin, 2006; Scuderi et al., 2011, 2012, 2013). It is established that neuroinflammation is directly linked to neural dysfunction and cell death, representing a primary cause of neurodegeneration (Block and Hong, 2005). In fact, over-release of pro-inflammatory cytokines by glia cells causes neuronal dysfunction and loss of synapses, which correlates with memory decline. These phenomena are believed to precede neuronal death. Thus, research focused on developing therapeutic strategies directed at controlling the prolonged and uncontrolled glia activation should be encouraged.

An uncommon opportunity to improve inflammation and neurodegeneration simultaneously is provided by compounds able to modulate histone acetylation/deacetylation, since they participate in brain immune control and neuroprotection, in addition to their well-known effects on the molecular mechanisms associated to senescence and metabolic syndromes. Mounting evidence indicates sirtuins (SIRTs) exert neuroprotective effects in several models of neurodegeneration (Outeiro et al., 2008; Tang and Chua, 2008; de Oliveira et al., 2010). SIRTs, a family of NAD^+^-dependent enzymes with seven isoforms identified (SIRT1-7), are implicated in the control of a variety of biological processes including transcriptional silencing, chromosomal stability, cell cycle progression, apoptosis, autophagy, metabolism, growth suppression, inflammation, and stress response (Gan and Mucke, 2008; Haigis and Sinclair, 2010).

Recent observations indicate both SIRT1 and SIRT2 regulate neuronal survival, but with divergent functional outcomes. Indeed, activation of SIRT1 mainly exerts neuroprotective actions, while SIRT2 fosters neurodegeneration. The reason for such opposite effect may be due to their different sub-cellular localization, which gives SIRT1 and SIRT2 distinct molecular targets (Harting and Knöll, 2010). It has been demonstrated that the overexpression of SIRT1 prevents neuronal death in tissue culture models of AD, amyotrophic lateral sclerosis, and polyglutamine toxicity, and it reduces hippocampal degeneration in a mouse model of AD (Kim et al., 2007; Li et al., 2007). Moreover, treatment with resveratrol (RSV), a polyphenolic compound acting as a pharmacological activator of SIRT1, is protective in a number of experimental neurodegeneration paradigms (Anekonda, 2006; Sun et al., 2010). Resveratrol, like other polyphenol compounds including curcumin, displays a plethora of actions, behaving as a potent antioxidant agent, increasing SUMOylation, and activating protein kinase C, all mechanisms able to counteract astrocyte reactivity and protect neurons (Jefremov et al., 2007; Hoppe et al., 2013; Menard et al., 2013). Finally, it has been observed that both SIRT1 overexpression and RSV treatment are able to significantly decrease the Aβ-induced activation of NF-κB, thus operating a simultaneous control on both neurodegeneration and neuroinflammation processes (Chen et al., 2005). Indeed, NF-κB is a transcription factor which controls the expression of gene products involved in key cellular signaling, including those associated to inflammatory and degenerative events. Post-mortem studies on cerebral cortices from AD patients have established a correlation between loss of SIRT1 and the accumulation of Aβ and hyperphosphorylated tau proteins (Julien et al., 2009). Growing evidence indicates that also SIRT2 is involved in regulating several brain processes including oligodendrocyte mitosis and differentiation, cytoskeletal dynamics necessary for trafficking, neurite outgrowth and synaptic remodeling. Unlike SIRT1, SIRT2 appears to promote neuronal death. In fact, blocking SIRT2 counteracted alpha synuclein toxicity in Parkinson’s disease models (Outeiro et al., 2007). However, less is known about the role of SIRT2 in AD.

On the basis of these considerations, we explored the effects of modulators of SIRTs on astrocyte activation and the subsequent inflammatory process. In particular our experiments focalized the ability of RSV, a SIRT1 activator, and AGK-2, a SIRT2-selective inhibitor, to control astrocyte activation and to suppress the production of pro-inflammatory mediators in primary rat astrocytes exposed to Aβ peptide. These findings suggest that either RSV or AGK-2 may be an effective agent for neurodegenerative diseases initiated or maintained by inflammatory processes.

## MATERIALS AND METHODS

### CELL CULTURES AND TREATMENTS

Newborn Sprague-Dawley rats (1 or 2 days old) were used to obtain primary astroglial cultures (Vairano et al., 2002; Scuderi et al., 2011). Briefly, brain cortices were homogenized and processed to obtain single cells. Astrocytes were cultured at a density of 3 × 106 cells/75-cm^2^ flask and incubated at 37°C in a humidified atmosphere containing 5% CO2. The culture medium used was DMEM supplemented with 5% inactivated fetal bovine serum, 100 IU/ml penicillin and 100 μg/ml streptomycin (all from Sigma–Aldrich, Milan, Italy), replaced 24 h after isolation and again one a week until astrocytes were grown to form a monolayer. Approximately 14–15 days after dissection, astrocytes were mechanically separated from microglia and oligodendrocytes. Obtained astrocytes were seeded onto 10-cm-diameter Petri dishes (1 × 106 cells/dish) or onto 24 well plates (1 x 105 cells/well). The monoclonal anti-glial fibrillary acidic protein (GFAP) was used to verify cell culture purity. Only cultures with more than 95% GFAP-positive cells were utilized. The 5% of non-astrocyte cells were microglia and oligodendrocytes.

All experiments were performed in accordance with the Italian Ministry of Health (DL 116/92), the Declaration of Helsinki, and the Guide for the Care and Use of Mammals in Neuroscience and Behavioral Research, and they were approved by the Institutional Animal Care and Use Committee at our institution.

Mature astrocytes were challenged with 0.23 μM Aβ 1-42 (Tocris Bioscience, Bristol, UK) in the presence or absence of the following substances: RSV (2 – 10 – 50 μM), a well-known SIRT1 activator, or AGK-2 (0.35 – 3.5 – 35 μM), a potent SIRT2-selective inhibitor (both from Sigma–Aldrich). After 24 (for viability and protein expression analyses) or 72 h (for proliferation assay) of treatment, astrocytes were collected for experiments. The concentration of the substances was chosen according to literature (Howitz et al., 2003; Outeiro et al., 2007; Scuderi et al., 2011, 2012).

### ANALYSIS OF ASTROCYTE VIABILITY BY NEUTRAL RED UPTAKE ASSAY

Astrocyte viability was evaluated 24 h after treatments by the neutral red uptake assay according to Repetto et al. (2008), with some modifications (Scuderi and Steardo, 2013). Cells were seeded in 24-well plates and treated as previously described. 24 h after treatments, the plates were incubated for 3 h at 37°C with a neutral red working solution (50 μg ml^-1^ in PBS 1X without calcium and magnesium, Sigma-Aldrich). The cells were washed and the dye removed from each well through a destain solution (ethanol:deionized water: glacial acetic acid, 50:49:1, v/v). The absorbance was read at 540 nm using a microplate spectrophotometer (Epoch, Bio Teck, Winooski, VT, USA). The values of treated cells were referred to control non-exposed cultures, and expressed as percentage variation.

### ANALYSIS OF ASTROCYTE PROLIFERATION BY TRYPAN BLUE ASSAY

Trypan blue exclusion assay was performed to monitor astrocyte proliferation 72 h after treatments. This method is based on the principle that living cells do not take up the dye, whereas dead cells do. To determine the number of cells and their viability using trypan blue, 20 μl of trypsinized and re-suspended cells were mixed with 20 μl of 0.4% solution of trypan blue dye (Sigma–Aldrich) for 1 min. Cells were immediately counted using a Bürker chamber with a light microscope. All counts were done using four technical duplicates of each sample.

### ANALYSIS OF PROTEIN EXPRESSION BY WESTERN BLOTTING

Western blot analyzes were performed on extracts of cell cultures challenged as previously described. 24 h after treatment, cells were detached from petri dishes and each pellet was suspended in ice-cold hypotonic lysis buffer containing NaCl 150 mM; Tris/HCl pH 7.5 50 mM; Triton X-100 1%; ethylenediaminetetraacetic acid [EDTA] 1 mM, supplemented with PMSF 1 mM, Aprotinin 10 μg/ml, Leupeptin 0,1 mM (Roche, Mannheim, Germany). After incubation for 40 min at +4°C, homogenates were centrifuged at 14000 rpm for 15 min and the supernatant removed and stored in aliquots at -80°C until use. Equivalent amounts (70 μg) of each sample calculated by Bradford assay were resolved on 12% acrylamide SDS-PAGE precast gels (Bio-Rad Laboratories). Proteins were transferred onto nitro-cellulose. Membranes were blocked with 5% wt/vol no-fat dry milk powder in Tris-buffered saline-Tween 0,1% (TBS-T) for 1 h before overnight incubation at 4°C with one of the following primary antibodies: rabbit anti-GFAP (1:50000, Abcam plc, Cambridge, UK), rabbit anti-S100B (1:1000, Epitomics, Burlingame, CA, USA), rabbit anti-inducible nitric oxide synthase (iNOS; 1:9000, Sigma–Aldrich), rabbit anti-cyclooxygenase-2 (COX-2; 1:1000, Cell Signaling Technology, MA, USA), rabbit anti-β-actin (1:1500, Santa Cruz Biotechnology, Santa Cruz, CA, USA). After being extensively washed in TBS-T, membranes were incubated for 1 h at 25 °C with the secondary horseradish peroxidase-conjugated antibody (HRP conjugated goat anti-rabbit IgG, 1:30000, Jackson Immunoresearch Europe, Suffolk, UK). The immunocomplexes were visualized using an ECL kit (Amersham, Bucks, UK). Protein expression was quantified by densitometric scanning of the X-ray films with a GS 700 Imaging Densitometer (Bio-Rad laboratories) and a computer program (ImageJ software v1.44p, NIH, USA).

### STATISTICAL ANALYSIS

Analysis was performed using GraphPad Prism (GraphPad Software, San Diego, CA, USA). Data were analyzed by one way analysis of variance (ANOVA) to determine statistical differences between experimental groups. Multiple comparisons were performed with Bonferroni’s test for *post hoc* analyzes. Differences between mean values were considered statistically significant when *p* < 0.05.

## RESULTS

### EFFECT OF RSV AND AGK-2 ON ASTROCYTE VIABILITY AND PROLIFERATION

**FIGURE 1:**
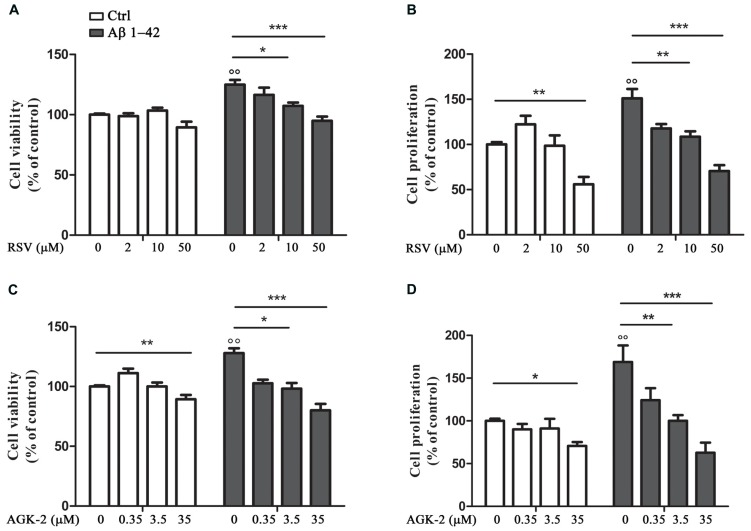
**Resveratrol (RSV) and AGK-2 affect astrocyte viability and proliferation induced by Aβ 1-42 challenge.** Cells were challenged with 0.23 μM Aβ 1–42 in the presence or absence of one of the following substances: RSV (2 – 10 – 50 μM), a potent SIRT1 activator; AGK-2 (0.35 – 3.5 – 35 μM), a selective SIRT2 inhibitor. 24 h later cell viability was assessed by neutral red uptake assay **(A,C)**. 74 h after treatments cell proliferation was evaluated by trypan blu assay **(B,D)**. Results are expressed as cell viability-fold increase versus unchallenged (open bars) or Aβ-challenged cells (black bars). Results are the mean ± SEM of four experiments in triplicate. Statistical analysis was performed by one-way ANOVA followed by Bonferroni multiple comparison test. *p* < 0.01 Aβ-challenged versus unchallenged cells; *p < 0.05; **p < 0.01; ***p < 0.001 for multiple comparison among groups.

First of all, we decided to perform experiments to assess the effect of the SIRT modulators on astrocyte viability and proliferation after Aβ 1-42 challenge. In fact, it has been already demonstrated that Aβ peptides are able to affect cell viability and to induce astrocyte proliferation (Allaman et al., 2010; Scuderi et al., 2012). Our results highlighted a significant increase in cell viability after 24 h treatment with Aβ 1-42 (**Figures 1A,C**, *p* < 0.01). RSV and AGK-2 were able to reduce this effect at the two higher concentrations used (**Figures 1A,C**). In addition, we found a reduction in cell viability after treatment with AGK-2 at the concentration of 35 μM on un-stimulated cells, indicating a cytotoxic effect (**Figure 1C**). Trypan blue experiments revealed a significant astrocyte proliferation after 72 h treatment with Aβ 1-42 (**Figures 1B,D**, *p* < 0.01). Once again, both RSV and AGK-2 significantly controlled such increase at the two higher concentrations used. Surprisingly, RSV 50 μM and AGK-2 35 μM caused a reduction in proliferation rate also in un-challenged astrocytes (**Figures 1B,D**).

### EFFECT OF RSV AND AGK-2 ON ASTROCYTE ACTIVATION

**FIGURE 2:**
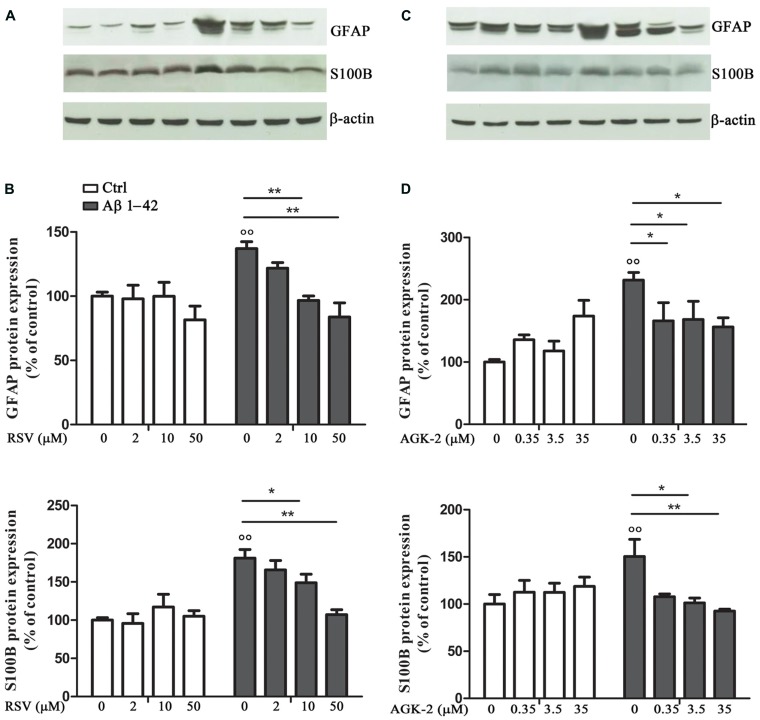
**Effect of RSV and AGK-2 on GFAP and S100B expression.** 24 h after treatments, astrocytes were lysated and protein expression was evaluated. Representative Western blots for GFAP and S100B proteins in lysates from astrocytes challenged with Aβ 1-42 (0.23 μM) in the presence of RSV (2 – 10 – 50 μM; **A**) or AGK-2 (0.35 – 3.5 – 35 μM; **C**). Densitometric analyzes normalized to β-actin loading controls (**B,D** for RSV and AGK-2, respectively). Results are the mean ± SEM of four experiments in triplicate. Statistical analysis was performed by one-way ANOVA followed by Bonferroni multiple comparison test. *p* < 0.01 Aβ-challenged versus unchallenged cells; *p < 0.05; **p < 0.01; for multiple comparison among groups.

In order to test the effect of RSV and AGK-2 on Aβ-induced astrogliosis, the expression of GFAP and S100B, specific markers of astrocyte activity, was explored. Reactive astrocytes display hypertrophied cell bodies and thickened processes exhibiting GFAP-immunoreactivity (O’Callaghan and Sriram, 2005; Olabarria et al., 2010). Using Western blot analysis, we observed a marked increase in the expression of GFAP after Aβ 1-42 challenge (*p* < 0.01; **Figure 2**). RSV was able to significantly attenuate such increase in a concentration dependent manner (**Figures 2A,B**). Likewise, the Aβ-induced GFAP overexpression was counteracted by AGK-2 at the three concentrations used (**Figures 2C,D**).

Similarly, the expression of S100B was investigated by Western blot. S100B is an astroglia-derived protein which acts as a neurotrophic factor and neuronal survival protein, even though the overproduction of S100B by activated astrocytes lead to further neurodegeneration. Elevated S100B levels are generally associated with a sustained reactive gliosis (Griffin, 2006; Donato and Heizmann, 2010). Results from cultured astrocytes showed a significant increase in S100B protein expression after Aβ 1-42 exposure (*p* < 0.01; **Figure 2**). Both RSV and AGK-2 controlled such increase. Also in this case, RSV exerted its effect in a concentration dependent manner (**Figures 2A,B**). Instead, all the AGK-2 concentrations completely abolished the Aβ-induced S100B increase (**Figures 2C,D**).

### EFFECT OF RSV AND AGK-2 ON INFLAMMATION

**FIGURE 3:**
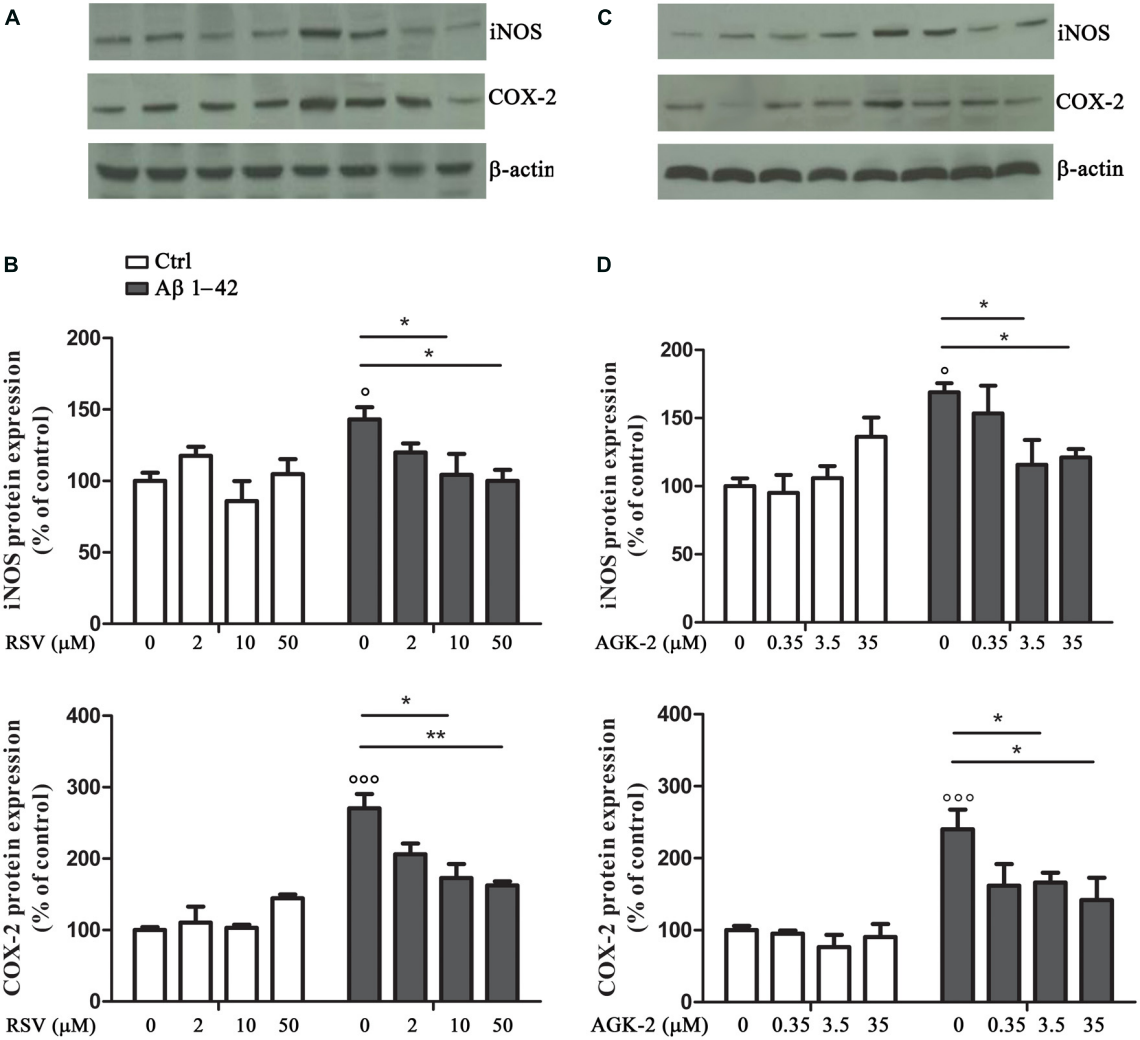
**Effect of RSV and AGK-2 on iNOS and COX-2 expression.** Astrocytes were treated with Aβ 1-42 (0.23 μM) in the presence of RSV (2 – 10 – 50 μM) or AGK-2 (0.35 – 3.5 – 35 μM). Western blot experiments were carried out 24 h after treatments. Representative immunoblots for iNOS and COX-2 proteins (**A,C** for RSV and AGK-2, respectively). Densitometric analyzes normalized to β-actin loading controls (**B,D** for RSV and AGK-2, respectively). Results are the mean ± SEM of four experiments in triplicate. Statistical analysis was performed by one-way ANOVA followed by Bonferroni multiple comparison test. *p* < 0.001 and *p* < 0.05 Aβ-challenged versus unchallenged cells; *p < 0.05; **p < 0.01; for multiple comparison among groups.

Another set of experiments was aimed at assessing the effect of RSV and AGK-2 on the production of inflammatory factors induced by Aβ 1-42 challenge. In fact, astrocyte activation is linked to the production of pro-inflammatory mediators which, in turn, stimulate gliosis and can kill neighboring neurons (Mrak and Griffin, 2001; Ferreira et al., 2014). Treatment with Aβ 1-42 resulted in an increase in iNOS expression, as determined by Western blot analysis (**Figure 3**; *p* < 0.05). Interestingly, this observed effect was reduced by both RSV and AGK-2 at the two higher concentrations used (**Figures 3A–D**). Parallel results were obtained with immunoblot experiments aimed at studying COX-2 expression. In fact, Aβ 1-42 significantly increased COX-2 protein expression (**Figure 3**; *p* < 0.05). Also in this case, both RSV and AGK-2 significantly decreased such effect at the two higher concentrations used (**Figures 3A–D**).

## DISCUSSION

The purpose of this study was to assess the efficacy of RSV, a well-known SIRT1 activator, and AGK-2, a potent SIRT2-selective inhibitor, in counteracting reactive gliosis, now considered one of the characteristic phenomena occurring in AD. AD leads to disability and death in a significant proportion of the world’s aged population (Alzheimer’s Association Report, 2013). However, the available treatments are limited and exert only symptomatic effects. Several promising drugs have recently failed to provide benefit, so there is urgent need to develop new, and hopefully more efficacious, drugs to affect AD course. To this attempt, in the last years researchers focused their attention on the role of reactive gliosis in the onset and progression of many neurodegenerative disorders, including AD. Produced results gave evidence that neuroinflammation and neurodegeneration mutually have a critical impact on AD course (Wyss-Coray and Rogers, 2012). For this reason, it is possible to assume that early combination of neuroprotective and anti-inflammatory treatments represents a particularly appropriate approach to AD (Di Filippo et al., 2008). Although neurodegenerative disorders have distinct clinical manifestations, many of the underlying pathogenic processes are similar (intra- or extracellular accumulation of misfolded proteins, cytoskeletal abnormalities, disruption of calcium homeostasis, mitochondrial dysfunction, and inflammation), and most of them are strongly influenced by and increased during aging. In particular, in both early- and late-onset sporadic AD, aging represents a major contributing factor for the disease development and progression, although the precise role remains still unclear. Transcriptional profiling studies revealed that expression of genes that play central roles in synaptic plasticity, vesicular transport and mitochondria function is reduced, whereas expression of genes encoding for stress, inflammatory or immune factors is increased in aged human frontal cortex (Lu et al., 2004). These findings implicate ongoing DNA damage, oxidative stress and inflammation as contributors to the functional decline occurring in age-related neurodegenerative diseases, including AD.

In this context, the discovery of SIRTs, indicated as class III histone deacetylases (HDACs), offers a close relationship between aging, metabolism and neurodegeneration, thereby representing an innovative target to develop therapeutic strategies (Outeiro et al., 2008). SIRTs play pleiotropic biological functions that range from repression of gene expression (through histone deacetylation) to regulation of cellular differentiation and/or apoptotic processes, from control of energetic cell metabolism to that of aging events. These enzymes have been extensively studied because of their involvement in mediating the effect of caloric restriction (CR) in fostering longevity and healthy aging. In addition, many data indicate that SIRTs are potentially able to delay neurodegenerative diseases related to senescence, including AD. (Michan and Sinclair, 2007). It has been demonstrated that CR reduces the content of Aβ in the temporal cortex of squirrel monkeys, and such effect is inversely linked to SIRT1 expression in the same brain region (Qin et al., 2006a). Moreover, in a transgenic mouse model of AD, the same authors previously demonstrated that CR antagonizes Aβ neuropathology by increasing the SIRT1 and NAD^+^/nicotinamide ratio (Qin et al., 2006b). Recently, SIRT2 inhibition has been proposed as a promising therapeutic strategy to achieve neuroprotection in *in vitro* and *in vivo* models of Parkinson’s and Huntington’s diseases (Outeiro et al., 2007; Luthi-Carter et al., 2010). Moreover, Spires-Jones et al. (2012) demonstrated that inhibition of SIRT2 is a safe and promising neuroprotective agent in both tau-associated frontotemporal dementia and AD.

It is recognized that Aβ affects cell viability and proliferation (Allaman et al., 2010; Scuderi et al., 2012). It is possible to speculate that these Aβ actions are due to its ability to enhance astrocyte metabolism turning on morpho-functional changes in such cells (Verkhratsky and Butt, 2007). Interestingly, our experiments highlighted alterations in astrocyte viability and proliferation after Aβ 1-42 challenge, and both RSV and AGK-2 markedly controlled these effects. SIRTs are considered as sensors of cell metabolic state because they finely modulate physiological processes. For this reason it is important to establish the appropriate concentrations to avoid dangerous unwanted consequences. In fact, in our conditions, we found that the highest concentrations used of both RSV and AGK-2 caused cytotoxic effects.

As a consequence of exogenous insults, glial cells lost their physiological functions and acquire a reactive phenotype, characterized by profound morphological and functional alterations, such GFAP and S100B overexpression (O’Callaghan and Sriram, 2005; Donato et al., 2013). In our model, we detected marked alteration of both these proteins. In fact, Western blot analysis showed that astrocytes express higher GFAP and S100B protein levels after Aβ challenge. Interestingly, RSV and AGK-2 negatively modulated the expression of both GFAP and S100B.

As mentioned before, the direct correlation between the Aβ-induced toxicity and the production of pro-inflammatory mediators prompted us to investigate the expression of the two main inducible enzymes related to inflammation, iNOS and COX-2. In our experimental condition, we highlighted the existence of an inflammatory state induced by Aβ 1-42 treatment, as detected by the increased expression of both iNOS and COX-2. The alteration of these two proteins was significantly blunted by RSV and AGK-2, indicating a key role in regulating astrogliosis and important astrocyte changes, which contribute to disease progression. In the current study it was observed that SIRT1 and SIRT2 can represent promising targets, whose manipulation could prevent over-activation of neuroglia upon pro-inflammatory stimulation. These data suggest a SIRT-dependent mechanism to restrain detrimental effects of excessive astrocyte activation. Moreover, the findings bear major implications in the context of several inflammatory conditions of the central nervous system where astroglia are known to mediate deleterious consequences. In conclusion, the results of the present study provide evidence that SIRT modulation can represent a strategy to counteract reactive gliosis, and suggest new avenues to walk for the discovery of novel and promising therapy for AD.

## AUTHOR CONTRIBUTIONS

Caterina Scuderi, Claudia Stecca, Bronzuoli M. Rosanna, Dante Rotili, Sergio Valente, Antonello Mai, Luca Steardo contributed to the work design, the acquisition and interpretation of data. Caterina Scuderi, Claudia Stecca, Bronzuoli M. Rosanna, Dante Rotili, Sergio Valente drafted the manuscript and revised it. Antonello Mai and Luca Steardo approved the final version of the manuscript. Caterina Scuderi, Claudia Stecca, Bronzuoli M. Rosanna, Dante Rotili, Sergio Valente, Antonello Mai, Luca Steardo ensure that questions related to the accuracy or integrity of any part of the work are appropriately investigated and resolved.

## Conflict of Interest Statement

The authors declare that the research was conducted in the absence of any commercial or financial relationships that could be construed as a potential conflict of interest.
